# Supplementary material for: Heat adaptation of phage T7 under an extended genetic code
Source: Virus Evol. 2021 Dec 1;7(2):veab100. doi: 10.1093/ve/veab100 (PMC8923235; doi:10.1093/ve/veab100)
Supplement: veab100_Supp [file veab100_supp.zip › Sequencing_Analysis.zip!/Sequencing_Analysis/S1/evidence/JC_SIDE_1_53.html]

BRESEQ :: Evidence


| New junction evidence | | | | | | | | | | | |
| --- | --- | --- | --- | --- | --- | --- | --- | --- | --- | --- | --- |
|  | | seq id | position | reads (cov) | reads (cov) | score | skew | freq | annotation | gene | product |
| \* | ? | NC\_001604.1 | = 9895 | 4719 (1.120) | 64 (0.020) | 49/592 | 27.8 | 1.3% | intergenic (–/–) | *–/–* | –/– |
|  | ? | NC\_001604.1 | 10215 = | 4867 (1.180) |  |  |  |  | intergenic (–/–) | *–/–* | –/– |
| Rejected: Coverage evenness skew score above cutoff. | | | | | | | | | | | |
| Rejected: Frequency below/above cutoff threshold. | | | | | | | | | | | |

Only 100 of 4735 total aligned reads displayed.  

`CTCTAAGCTGAAAGTTAAATATTCTCTGGTTCCATACAAGTGGAACACTGCTGTAGGTGCGAGCGTTAAGCTGCAACTGGAATCCGTGATGCTGGTCGAACTGGCTACCTTTGGTGGCGGTGAAGACGATTGGGCTGACGAAGTTGAAGAGAACGGCTATGTTGCCTCTGGTTCTGCCAAAGCGAGCAAACCACGCGACGAAGAAAGCTGGGACGAAGACGACGAAGAGTCCGAGGAAGCAGACGAAGACGGAGACTTCTAAGTGGAACTGCGGGAGAAAATCCTTGAGCGAATCAAGGTGACTTCCTCTGGGTGTTGGGAGTGGCAGGGCGCTACGAACAATAAAGGGTACGGGCAGGTGTGGTGCAGCAATACCGGAAAGGTTGTCTACTGTCATCGCGTAATGTCTAATGCTCCGAAAGGTTCTACCGTCCTGCACTCCTGTGATAATCCATTATGTTGTAACCCTGAACACCTATCCATAGGAACTCCAAAAGAGAACTCCACTGACATGGTAAATAAGGGTCGCTCACACAAGGGGTATAAACTTTCAGACGAAGACGTAATGGCAATCATGGAGTCCAGCGAGTCCAATGTATCC  >  NC_001604.1/9595‑10195`  

                                                                                                                                                                                                                                                                                                            `|`                                                                                                                                                                                                                                                                                                              
            `aGTTAAATATTCTCTGGTTCCATACAAGTGGAACACTGCTGTAGGTGCGAGCGTTAAGCTGCAACTGGAATCCGTGATGCTGGTCGAACTGGCTACCTTTGGTGGCGGTGAAGACGATTTGGCTGACGAAGTTGAAGAGAACGGCTATGTTGCCTCTGGTTCTGCCAAAGCGAGCAAACCACGCGACGAAGAAAGCTGGGACGAAGACGACGAAGAGTCCGAGGAAGCAGACGAAGACGGAGACTTCTAAGTGGAACTGCGGGAGAAAATCCTTGAGCGAATCAAGGTGACTTCCTCTggg                                                                                                                                                                                                                                                                                                  <  1:557477/301‑1 (MQ=255)`  
            `aGTTAAATATTCTCTGGTTCCATACAAGTGGAACACTGCTGTAGGTGCGAGCGTTAAGCGGCAACTGGAATCCGTGATGCTGGTCGAACTGGCTACCTTTGGTGGCGGTGAAGACGATTGGGCTGACGAAGTTGAAGAGAACGGCTATGTTGCCTCTGGTTCTGCCAAAGCGAGCAAACCACGCGACGAAGAAAGCTGGGACGAAGACGACGAAGAGTCCGAGGAAGCAGACGAAGACGGAGACTTCTAAGTGGAACTGCGGGAGAAAATCCTTGAGCGAATCAAGGTGACTTCCTCTggg                                                                                                                                                                                                                                                                                                  <  1:34535/301‑1 (MQ=255)`  
              `ttAAATATTCTCTGGTTCCATACAAGTGGAACACTGCTGTAGGTGCGAGCGTTAAGCTGCAACTGGAATCCGTGATGCTGGTCGAACTGGCTACCTTTGGTGGCGGTGAAGACGATTGGGCTGACGAAGTTGAAGAGAACGGCTATGTTGCCTCTGGTTCTGCCAAAGCGAGCACACCACGCGACGAAGCAAGCTGGGACGAAGACGACGAAGAGTCCGAGGAAGCAGACGCAGACGGAGACTTCTAAGTGGAACTGCGGGAGAAAATCCTTGAGCGAATCAAGGTGACTTCCTCTGGgtg                                                                                                                                                                                                                                                                                                >  1:581850/1‑301 (MQ=255)`  
                   `tatatgagacagccATACAATTGGAACACTGCTGTAGGTGCGAGCGTTAAGCTGCAACTGGAATCCGTGATGCTGGTCGAACTGTCTACCTTTGGTGGCGGTGAAGACGATTGGGCTGACGAAGTTGAAGAGAACGGCTATGTTGCCTCTGGTTCTGCCAAAGCGAGCAAACCACGCGACGAAGAAAGCTGGGACGAAGACGACGAAGAGTCCGAGGAAGCAGACGAAGACGGAGACTTCTAAGTGGAACTGCGGGAGAAAATCCTTGAGCGAATCAAGGTGACTTCCTCTGGGTGTTggt                                                                                                                                                                                                                                                                                           <  1:3892/289‑2 (MQ=255)`  
                   `tataagggccagccATACAAGTGGAACACTGCTGTAGGTGCGAGCGTTAAGCTGCAACTGGAATCCGTGATGCTGGTCGAACTGGCTACCTTTGGTGGCGGTGAAGACGATTGGGCTGACGAAGTTGAAGAGAACGGCTATGTTGCCTCTGGTTCTGCCAAAGCGAGCAAACCACGCGACGAAGAAAGCTGGGACGAAGACGACGAAGAGTCCGAGGAAGCAGACGAAGACGGAGACTTCTAAGTGGAACTGCGGGAGAAAATCCTTGAGCGAATCAAGGTGACTTCCTCTGGGTGTTggg                                                                                                                                                                                                                                                                                           <  1:527216/289‑1 (MQ=255)`  
                   `tatTCTCTGGTTCCATACAAGTGGAACACTGCTGTAGGTGCGAGCGTTAAGCTGCAACTGGAATCCGTGATGCTGGTCGAACTGGCTACCTTTGGTGGCGGTGAAGACGATTGGGCTGACGAAGTTGAAGAGAACGGCTATGTTGCCTCTGGTTCTGCCAAAGCGAGCAAACCACGCGACGAAGAAAGCTGGGACGAAGACGACGAAGAGTCCGAGGAAGCAGACGAAGACGGAGACTTCTAAGTGGAACTGCGGGAGAAAATCCTTGAGCGAATCAAGGTGACTTCCTCTGGGTGTTggg                                                                                                                                                                                                                                                                                           <  1:333214/301‑1 (MQ=255)`  
                    `atTCTCTGGTTCCATACAAGTGGAACACTGCTGTAGGTGCGAGCGTTAAGCTGCAACTGGAATCCGTGATGCTGGTCGAACTGGCTACCTTTGGTGGCGGTGAAGACGATTGGGCTGACGAAGTTGAAGAGTACGGCTATGTTGCCTCTGGTTCTGCCAAAGCGAGCAAACCACGCGACGAAGAAAGCTGGGACGAAGACGACGAAGAGTCCGAGGAAGCATACGAAGACGGAGACTTCTAAGTGGAACTGCGGGAGAAAATCCTTGAGCGAATCAAGGTGACTTCCTCTgggctgtctct                                                                                                                                                                                                                                                                                          >  1:322020/1‑293 (MQ=255)`  
                      `tctcTGGTTCCATACAAGTGGAACACTGCTGTAGGTGCGAGCGTTAAGCTGAAACTGGAATCCGTGATGCTGGTCGAACTGGCTACCTTTGGTGGCGGTGAAGACGATTGGGCTGACGAAGTTGAAGAGAACGGATATGTTGCCTCTGGTTCTGCCAAAGCGAGCAAACCACGCGACGAAGAAAGCTGGGACGAAGACGACGAAGAGTCCGAGGAAGCAGACGAAGACGGAGACTTCTAAGTGGAACTGCGGGAGAAAATCCTTGAGCGAATCAAGGTGACTTCCTCTGGGTGTTGGGAGt                                                                                                                                                                                                                                                                                        <  1:654216/301‑1 (MQ=255)`  
                            `gTTCCATACAAGTGGAACACTGCTGTAGGTGCGAGCGTTAAGCTGCAACTGGAATCCGTGATGCTGGTCGAACTGGCTACCTTTGGTGGCGGTGAAGACGATTGGGCTGACGAAGTTGAAGAGAACGGCTATGTTGCCTCTGGTTCTGCCAAAGCGAGCAAACCACGCGACGAAGAAAGCTGGGACGAAGACGACGAAGAGTCCGAGGAAGCAGACGAAGACGGAGACTTCTAAGTGGAACTGCGGGAGAAAATCCTTGAGCGAATCAAGGTGACTTCCTCTGGGTGTTgggctgtctctt                                                                                                                                                                                                                                                                                  >  1:148993/1‑292 (MQ=255)`  
                               `ccATACAAGTGGAACACTGCTGTAGGTGCGAGCGTTAAGCTGCAACTGGAATCCGTGATGCTGGTCGAACTGGCTACCTTTGGTGGCGGTGAAGACGATTGGGCTGACGAAGTTGAAGAGAACGGCTATGTTGCCTCTGGTTCTGCCAAGGCGAGCAAACCACGCGACGAAGAAAGCTGTGACGAAGACGACGAAGAGTCCGAGGAAGCAGACGAAGACGGAGACTTCTAAGTGGAACTGCGGGAGAAAATCCTTGAGCGAATCAAGGTGACTTCCTCTGGGTGTTGGGACTgtctcttat                                                                                                                                                                                                                                                                               >  1:463454/1‑293 (MQ=255)`  
                                     `aaGTGGAACACTGCTGTAGGTGCGAGCGTTAAGCTGCAACTGGAATCCGTGATGCTGGTCGAACTGGCTACCTTTGGTGGCGGTGAAGACGATTGGGCTGACGAAGTTGAAGAGAACGGCTATGTTGCCTCTGGTTCTGCCAAAGCGAGCAAACCACGCGACGAAGAAAGCTGGGACGAAGACGACGAAGAGTCCGAGGAAGCAGACGAAGACGGAGACTTCTAAGTGGAACTGCGGGAGAAAATCCTTGAGCGAATCAAGGTGACTTCCTCTGGGTGTTGGGAGTGGCAGGGCGCTACGa                                                                                                                                                                                                                                                                         >  1:433277/1‑301 (MQ=255)`  
                                          `gAACACTGCTGTAGGTGCGAGCGTTAAGCTGCAACTGGAATCCGTGATGCTGGTCGAACTGGCTACCTTTGGTGGCGGTGAAGACGATTGGGCTGACGAAGTTGAAGAGAACGGCTATGTTGCCTCTGGTTCTGCCAAAGCGAGCAAACCACGCGACGAAGAAAGCTGGGACGAAGACGACGAAGAGTCCGAGGAAGCAGACGAAGACGGAGACTTCTAAGTGGAACTGAGGGAGAAAATCCTTGAGCGAATCAAGGTGACGTCCTATGGGAGTTGGGAGTGCAAGGGCGCGACGAaaaaa                                                                                                                                                                                                                                                                    >  1:445720/1‑300 (MQ=255)`  
                                            `aaacaggtgTAGGGGCGAGCGGTGATCTGCAACTGGAATCAGTGATGCGGGTTGAACTGATTAACTTTGGAGGCGGTGAAGACGATAGGGCAGACGAAGTGAAAGATAACGGCTATGGTGCCACTGGTACTGCCAAAGCGAGCAAACCACGCGACGAAGGAAGCTGGGAAGAAGACGACGAAGAGAGGGAGGAAGAAGACGAAGACGGAGAGATATAAGTGGAACTGCGGGAGAAAATACTTGAGCGAATCAAGGTGACGACCTCTGGGTGTTGGGAGTGGCAGGGCGCTACGAACAATaa                                                                                                                                                                                                                                                                  <  1:618809/294‑1 (MQ=255)`  
                                               `ctgctgTAGGTGCGAGCGTTAAGCTGCAACTGGAATCCGTGATGCTGGTCGAACTGGCTACCTTTGGTGGCGGTGAAGACGATTGGGCTGACGAAGTTGAAGAGAACGGCTATGTTGCCTCTGGTTCTGCCAAAGCGAGCAAACCACGCGACGAAGAAAGCTGGGACGAAGACGACGAAGAGTCCGAGGAAGCAGACGAAGACGGAGACTTCTAAGTGGAACTGCGGGAGAAAATCCTTGAGCGAATCAAGGTGACTTCCTCTGGGTGTTGGGAGTGGCAGGGCGCTACGAACAATAAAgg                                                                                                                                                                                                                                                               >  1:551382/1‑301 (MQ=255)`  
                                               `ctgctgTAGGTGCGAGCGTTAAGCTGCAACTGGAATCCGTGATGCTGGTCGAACTGGCTACCTTTGGTGGCGGTGAAGACGATTGGGCTGACGAAGTTGAAGAGAACGGCTATGTTGCCTCTGGTTCTGCCAAAGCGAGCAAACCACGCGACGAAGAAAGCTGGGACGAAGACGACGAAGAGTCCGAGGAAGCAGACGAAGACGGAGACTTCTAAGTGGAACTGCGGGAGAAAATCCTTGAGCGAATCAAGGTGACTTCCTCTGGGTGTTGGGAGTGGCAGGGCGCTACGAACAATAAAgg                                                                                                                                                                                                                                                               >  1:612852/1‑301 (MQ=255)`  
                                                `tgctgTAGGTGGTAGCGTTAAGCTGCAACTGGAATCCGTGATGCTGGTCGAACTGGCTACCTTTGGTGGCGGTGAAGACGATTGGGCTGACGAAGTTGAAGAGAACGGCTATGTTGCCTCTGGTTCTGCCAAAGCGAGCAAACCACGCGACGAAGAAAGCTGGGACGAAGACGACGAAGAGTCCGAGGAAGCAGACGAAGACGGAGACTTCTAAGTGGAACTGCGGGAGAAAATCCTTGAGCGAATCAAGGTGACTTCCTCTGGGTGTTGGGAGTGGCAGGGCGCTACGAACAATAAAggg                                                                                                                                                                                                                                                              <  1:47037/301‑1 (MQ=255)`  
                                                `agctgTAGGTGCGAGCGTTAAGCTGCAAATGGAATCCGTGATGCTGGTCGAACTGGCTACCTTTGGTGGCGGTGAAGACGATTGGGCTGACGAAGTTGAAGAGAACGGCTATGTTGCCTCTGGTTCTGCCAAAGCGAGCAAACCACGCGACGAAGAAAGCTGGGACGAAGACGACGAAGAGTCCGAGGAAGCAGACGAAGACGGAGACTTCTAAGTGGAACTGCGGGAGAAAATCCTTGAGCGAATCAAGGTGACTTCCTCTGGGTGTTGGGAGTGGCAGGGCGCTACGAACAATAAAggg                                                                                                                                                                                                                                                              <  1:168759/300‑1 (MQ=255)`  
                                                 `gctgTAGGTGCGAGCGTTAAGCTGCAACTGGAATCCGTGATGCTGGTCGAACTGGCTACCTTTGGTGGCGGTGAAGACGATTGGGCTGACGAAGTTGAAGAGAACGGCTATGTTGCCTCTGGTTCTGCCAAAGCGAGCAAACCACGCGACGAAGAAAGCTGGGACGAAGACGACGAAGAGTCCGAGGAAGCAGACGAAGACGGAGACTTCTAAGTGGAACTGCGGGAGAAAATCCTTGAGCGAATCAAGGTGACTTCCTCTGGGTGTTGGGAGTGGCAGGGCGCTACGAACAATAAAgggc                                                                                                                                                                                                                                                             >  1:90503/1‑300 (MQ=255)`  
                                                      `aGGTGCGAGCGTTAAGCTGCAACTGGAATCCGTGATGCGGGTCGAACTGGCTACCTTTGGTGGCGGTGAAGACGATTGGGCTGACGAAGTTGAAGAGAACGGCTATGTTGCCTCTGGTTCTGCAAAAGCGAGCAAACCACGCGACGAAGAAAGCTGGGACGAAGACGACGAAGAGTCCGAGGAAGAAGACGAAGACGGAGACTTCTAAGTGGAACTGCGGGAGAAAATCCTTGAGCGAATCAAGGTGACTTCCGCTGGGTGTTGGGAGTGGCAGGGCGCTACGAACAATAAAGGGTACggg                                                                                                                                                                                                                                                        <  1:243383/301‑1 (MQ=255)`  
                                                       `ggggCGAGAGTTAAGCTGCAACTGGAATCCGTGATGCTGGTCGAACTGGCTACCTTTGGTGGCGGTGAAGACGATTGGGCTGACGAAGTTGAAGAGAACGGCTATGTTGCCTCTGGTTCTGCCAAAGCGAGCAAACCACGCGACGAAGAAAGCTGGGACGAAGACGACGAAGAGTCCGAGGAAGCAGACGAAGACGGAGACTTCTAAGTGGAACTGCGGGAGAAAATCCTTGAGCGAATCAAGGTGACTTCCTCTGGGTGTTGGGAGTGGCAGGGCGCTACGAACAATAAAGGGTACGGGc                                                                                                                                                                                                                                                       <  1:83441/298‑1 (MQ=255)`  
                                                         `tGCGAGCGTTAAGCTGCAACTGGAATCCGTGATGCTGGTCGAACTGGCTACCTTTGGTGGCGGTGAAGACGATTGGGCTGACGAAGTTGAAGAGAACGGCTATGTTGCCTCTGGTTCTGCCAAAGCGAGCAAACCACGCGACGAAGAAAGCTGGGACGAAGACGACGAAGAGTCCGAGGAAGCAGACGAAGACGGAGACTTCTAAGTGGAACTGCGGGAGAAAATCCTTGAGCGAATCAAGGTGACTTCCTCTGGGTGTTGGGAGTGGCAGGGCGCTACGAACAATaactgtctcttataa                                                                                                                                                                                                                                                     >  1:174304/1‑288 (MQ=255)`  
                                                             `aGCGTTAAGCTGCAACTGGAATCCGTGATGCGGGTCGAACTGGCTAACGTTGGTGGCGGTGAAGACGATTGGGCTGACGAAGTTGAAGAGAACGGCTATGTTGCCTCTGGTTCTGCCAAAGCGAGCAAACCACGCGACGAAGAAAGCTGGGACGAAGACGACGAAGAGTCCGAGGAAGCAGACGAAGACGGAGACTTCTAAGTGGAACTGCGGGAGAAAATCCTTGAGCGAATCAAGGTGACTTCCTCTGGGTGTTGGGAGTGGCAGGGCGCTACGAACAATAAAGGGTACGGGCAGgtgt                                                                                                                                                                                                                                                 <  1:357244/301‑1 (MQ=255)`  
                                                              `gCGTTAAGCTGCAACTGGAATCCGTGATGCTGGTCGAACTGGCTACCTTTGGTGGCGGTGAAGACGATTGGGCTGACGAAGTTGAAGAGAACGGCTATGTTGCCTCTGGTTCTGCCAAAGCGAGCAAACCACGAGACGAAGAAAGCTGGGACGAAGACGACGAAGAGTCCGAGGAAGCAGACGAAGACGGAGACTTCTAAGTGGAACTGCGGGAGAAAATCCTTGAGCGAATCAAGGTGACTTCCTCTGGGTGTTGGGAGTGGCAGGGCGCTACGAAcctgtctcttatacacatctccga                                                                                                                                                                                                                                                >  1:401532/1‑278 (MQ=255)`  
                                                                       `ggcagatGGAATCCGTGATGCTGGTCGAACTGGCTACCTTTGGTGGCGGTGAAGACGATTGGGCTGACGAAGTTGAAGAGAACGGCTATGGTGCCTCTGGTTCTGCCAAAGCGAGCAAACCACGCGACGAAGAAAGCTGGGACGAAGACGACGAAGAGTCCGAGGAAGCAGACGAAGACGGAGACTTCTAAGTGGAACTGCGGGAGAAAATCCTTGAGCGAATCAAGGTGACTTCCTCTGGGTGTTGGGAGTGGCAGGGCGCTACGAACAATAAAGGGTACGGGCAGGTGTGGTGCAGCaa                                                                                                                                                                                                                                       <  1:660032/295‑1 (MQ=255)`  
                                                                        `gCAACTGGAATCCGTGATGCTGGTCGAACTGGCTACCTTTGGTGGCGGTGAAGACGATTGGGCTGACGAAGTTGAAGAGAACGGCTATGTTGCCTCTGGTTCTGCCAAAGCGAGCAAACCACGCGACGAAGAAAGCTGGGACGAAGACGACGAAGAGTCCGAGGAAGCAGACGAAGACGGAGACTTCTAAGTGGAACTGCGGGAGAAAATCCTTGAGCGAATCAAGGTGACTTCCTCTGGGTGTTGGGAGTGGCCGGGCGCTACGAACAATAAAGGGTACGGGCAGGAGTGGTGCAGCAAt                                                                                                                                                                                                                                      >  1:123610/1‑301 (MQ=255)`  
                                                                        `gCAACTGGAATCCGTGATGCTGGTCGAACTGGCTACCTTTGGTGGCGGTGAAGACGATTGGGCTGACGAAGTTGAAGAGAACGGCTATGTTGCCTCTGGTTCTGCCAAAGCGAGCAAACCACGCGACGAAGAAAGCTGGGACGAAGACGACGAAGAGTCCGAGGAAGCAGACGAAGACGGAGACTTCTAAGTGGAACTGCGGGAGAAAATCCTTGAGCGAATCAAGGTGACTTCCTCTGGGTGTTGGGAGTGGCAGGGCGCTACGAACAATAAAGGGTACGGGCAGGTGTGGTGCAGCAAt                                                                                                                                                                                                                                      >  1:193547/1‑301 (MQ=255)`  
                                                                           `aCTGGAATCCGTGATGCTGGTCGAACTGGCTACCTTTGGTGGCGGTGAAGACGATTGGGCTGACGAAGTTGAAGAGAACGGCTATGTTGCCTCTGGTTCTGCCAAAGCGAGCAAAGCACGCGACGAAGAAAGCTGGGACGAAGACAACGCAGAGTCCGAGGAAGCAGACGAAGACGGAGACTTCTAAGAGGAAGTTCGAGAGAAAATCCTTGACCGAAGCAAGGTGACTTCATCTGGGCGGTGGGAGTGGCAGGACGCTACGAACAATCAAGGGTACGGGCAGGtgcagtgcaggcactgt                                                                                                                                                                                                                                   >  1:503175/1‑294 (MQ=255)`  
                                                                               `gAATCCGTGATGCTGGTCGAACGGGCTACCTTTGGTGGCGGTGAAGACGATTGGGCTGACGAAGTTGAAGAGAACGGCTATGTTGCCTCTGGTTCTGCCAAAGCGAGCAAACCACGCGACGAAGAAAGCTGGGACGAAGACGACGAAGAGTCCGAGGAAGCAGACGAAGACGGAGACTTCTAAGTGGAACTGCGGGAGAAAATCCTTGAGCGAATCAAGGTGACTTCCTCTGGGTGTTGGGAGTGGCAGGGCGCTACGAACAATAAAGGGTACGGGCAGGTGTGGTGCAGCAATACCGGaa                                                                                                                                                                                                                               <  1:642658/301‑1 (MQ=255)`  
                                                                                `aaTCCGTGATGCTGGTCGAACTGGCTACCTTTGGTGGCGGTGAAGACGATTGGGCTGACGAAGTTGAAGAGAACGGCTATGTTGCCTCTGGTTCTGCCAAAGCGAGCAAACCACGCGACGAAGAAAGCTGGGACGAAGACGACGAAGAGTCCGAGGAAGCATACGAAGACGGAGACTTCTAAGTGGAACTGCGGGAGAAAATCCTTGAGCGAATCAAGGTGACTTCCTCTGGGTGTTGGGAGTGGCAGGGCGCTACGAACAATAAAGGGTACGGGCAGGTGTGGTGCAGCAATACCGGaaa                                                                                                                                                                                                                              >  1:134519/1‑301 (MQ=255)`  
                                                                                   `ccGTGATGCTGGTCGAACTGGCTACCTTTGGTGGCGGTGAAGACGATTGGGCTGACGAAGTTGAAGAGCACGGCTATGTTGCCTCTGGTTCTGCCAACGCGAGCAAACCACGCGACGAAGAAAGCTTGAACGAAGACGACGAAGAGTCCGAGGAAGCAGACGAAGACGGAGACTTCTAAGTGGAACTGCGGGAGAAAATCCTTGAGCGAATCAAGGTGACTTCCTCTGGGTGTTGGGAGTGGCAGGGCGCTACGAACAATAAAGGGTACGGGCAGGTGTGGTGCAGCAATACCGGAAAGGt                                                                                                                                                                                                                           >  1:72323/1‑301 (MQ=255)`  
                                                                                   `ccGTGATGCTGGTCGAACTGGCTACCTTTGGTGGCGGTGAAGACGATTGGGCTGACGAAGTTGAAGAGAACGGCTATGTTGCCTCTGGTTCTGCCAAAGCGAGCAAACCACGCGACGAAGAAAGCTGGGACGAAGACGACGAAGAGTCCGAGGAAGCAGACGAAGACGGAGACTTCTACGTGGAACTGCGGGAGAAAATCCTTGAGCGAATCAAGGTGACTTCCTCTGGGTGTCGGGAGTGGCAGGGAGCTACGAACAATAAAGGGTACGGGCAGGTGTGGTGCAGCAATACCGGaactgt                                                                                                                                                                                                                           >  1:304577/1‑297 (MQ=255)`  
                                                                                   `ccGTGATGCTGCTCGAACTGGCTACCTTTGGTGACGGTGAAGACGATTGGGCTGACGAAGTTGAAGAGAGCGGCTATGTTGCCTCTGGGTCTGCCAAAGCGAGCAAACCACGCGACGAAGAAAGCTGGGACGAAGACGACGAAAAGTCCGAGGAAGCAGACGAAGACGGAGACTTCTAAGTGGAACTGCGGGAGAAAATCCTTGTGCGAATCAAGGTGACTTCCTCTGGGTGTTGGGAGTGGCAGGGCGCTACGAACAATAAAGGGTACGGGCAGGTGTGGTGCAGCAATACCGGAAAGGt                                                                                                                                                                                                                           <  1:539605/301‑1 (MQ=255)`  
                                                                                          `gCTGGTCGAACTGGGTAACTTTGGTGGCGGTGAAGACGATTGGGCTGACGAAGTTGAAGAGAACGGCTATGTTGCCTCTGGTTCTGCCAAAGCGAGCAAACCACGCGACGAAGAAAGCTGGGACGAAGACGACGAAGAGTCCGAGGAAGCAGACGAAGACGGAGACTTCTAAGTGGAACTGCGGGAGAAAATCCTTGAGCGAATCAAGGTGACTTCCTCTGGGTGTTGGGAGTGGCAGGGCGCTACGAACAATAAAGGGTACGGGCAGGTGTGGTGCAGCAATACCGGAAAGGTTGTCTAc                                                                                                                                                                                                                    <  1:212161/301‑1 (MQ=255)`  
                                                                                          `gCTGGTCGAACTGGCTACCTTTGGTGGCGGTGAAGACGATTGGGCTGACGAAGTTGAAGAGAACGGCTATGTTGCCTCTGGTTCTGCCAAAGCGAGCAAACCACGCGACGAAGAAAGCTGGGACGAAGACGACGAAGAGTCCGAGGAAGCAGACGAAGACGGAGACTTCTAAGTGGAACTGCGGGAGAAAATCCTTGAGCGAATCAAGGTGACTTCCTCTGGGTGTTGGGAGTGGCAGTGCGCTACGAACAATAAAGGGTACGGGCAGGTGTGGTGCAGCAATACCGGAAAGGTTGTCTAc                                                                                                                                                                                                                    <  1:648156/301‑1 (MQ=255)`  
                                                                                          `gCTGGTCGAACTGGCTACCTTTGGTGGCGGTGAAGACGATTGGGCTGACGAAGTTGAAGAGAACGGCTATGTTGCCTCTGGTTCTGCCAAAGCGAGCAAACCACGCGACGAAGAAAGCTGGGACGAAGACGACGAAGAGTCCGAGGAAGCAGACGAAGACGGAGACTTCTAAGTGGAACTGCGGGAGAAAATCCTTGAGCGAATCAAGGTGACTTCCTCTGGGTGTTGGGAGTGGCAGGGCGCTACGAACAATAAAGGGTACGGGCAGGTGTGGTGCAGCAATACCGGAAAGGTTGTCTAc                                                                                                                                                                                                                    <  1:484750/301‑1 (MQ=255)`  
                                                                                            `tGGTCGAACTGGCTACCTTTGGTGGCGGTGAAGACGATTGGGCTGACGAAGTTGAAGAGAACGGCTATGTTGCCTCTGGTTCTGCCAAAGCGAGCAAACCACGCGACGAAGAAAGCTGGGACGAAGACGACGAAGAGTCCGAGGAAGCAGACGAAGACGGAGACTTCTAAGTGGAACTGCGGGAGAAAATCCTTGAGCGAATCAAGGTGACTTCCTCTGGGTGTTGGGAGTGGCAGGGCGCTACGAACAATAAAGGGTACGGGCAGGTGTGGTGCAGCAATACCGGAAAGGTTGTCTACTg                                                                                                                                                                                                                  <  1:12877/301‑1 (MQ=255)`  
                                                                                             `gggcGAACTGGCTACCTTTGGTGGCGGTGAAGACGATTGGGCGGACGAAGTTGAAGAGAACGGCTATGTTGCCTCTGGTTCTGCCAAAGCGAGCAAACCACGCGACGAAGAAAGCTGGGACGAAGACGACGAAGAGTCCGAGGAAGCAGACGAAGACGGAGACTTCTAAGTGGAACTGCGGGAGAAAATCCTTGAGCGAATCAAGGTGACTTCCTCTGGGTGTTGGGAGTGGCAGGGCGCTACGAACAATAAAGGGTACGGGCAGGTGTGGTGCAGCAATACCGGAAAGGTTGTCTACTGt                                                                                                                                                                                                                 <  1:368643/298‑1 (MQ=255)`  
                                                                                                  `aaCTGGCTACCTTTGGTGGCGGTGAAGACGATTGGGCTGACGAAGTTGAAGAGAACGGCTATGTTGCCTCTGGTTCTGCCAAAGCGAGCAAACCACGCGACGAAGAAAGATGGGACGAAGACGACGAAGAGTCCGAGGAAGCAGACGAAGACGGAGACTTCTAAGTGGAACTGCGGGAGAAAATCCTTGAGCGAAACAAGGTGACTTCCTCTGGGTGTTGGGAGTGGCAGGGCGCTACGAACAATAAAGGGTACGGGCAGGTGTGGTGCAGCAATACCGGAACGGTAGTCTACTGTCATcg                                                                                                                                                                                                            >  1:238710/1‑301 (MQ=255)`  
                                                                                                    `cTGGCTACCTTTGGTGGCGGTGAAGACGATTGGGCTGACGAAGTTGAAGAGAACGGCTATGTTGCCTCTGGTTCTGCCAAAGCGAGCAAACCACGCGACGAAGAAAGCTGGGACGAAGACGACGAAGAGTCCGAGGAAGCAGACGAAGACGGAGACTTCTAAGTGGAACTGCGGTAGAAAATCCTTGAGCGAATCAAGGTGACTTCCTCTGGGTGTTGGGAGTGGCTGGGCGCTACGAACAATAACAGGTACGGGCAGGTGTGGTGCAGCAATACCGGAACGGTTGTCTACTGTCATcgcg                                                                                                                                                                                                          >  1:623966/1‑301 (MQ=255)`  
                                                                                                           `ccTTTGGTGGCGGTGAAGACGATTGGGCTGACGAAGTTGAAGAGAACGGCTATGTTGCCTCTGGTTCTGCCAAAGCGAGCAAACCACGCGACGAAGAAAGCTGGGACGAAGACGACGAAGAGTCCGAGGAAGCAGACGAAGACGGAGACTTATAAGTGGAACTGCGGGAGAAAATCCTTGAGCGAATCAAGGTGACTTCTTCTGGGTGTTGGGAGTGGCAGGGCGCTACGAACAATAAAGGGTAAGGGCAGGTGGGGTGCAGCAATACCGGAAAGGTTGTCTACTGTCATCGCGTAATGta                                                                                                                                                                                                   >  1:105665/1‑300 (MQ=255)`  
                                                                                                                 `gtggCGGTGAAGACGATTGGGCTGACGAAGTTGAAGAGAACGGCTATGTTGCCTCTGGTTCTGCCAAAGCGAGCAAACCACGCGACGAAGAAAGCTGGGACGAAGACGACGAAGAGTCCGAGGAAGCAGACGAAGACGGAGACTTCTAAGTGGAACTGCGGGAGAAAATCCTTGAGCGAATCAAGGTGACTTCCTCTGGGTGTTGGGAGTGGCAGGGCGCTACGAACAATAAAGGGTACGGGCAGGTGTGGTGCAGCAATACCGGAAAGGTTGTCTACTGTCATCGCGTAATGTCTAATGc                                                                                                                                                                                             <  1:545424/301‑1 (MQ=255)`  
                                                                                                                 `gatgtgtataagagacaggggCTGACGAAGTTGAAGAGAACGGCTATGTTGCCTCTGGTTCTGCCAAAGCGAGCAAACCACGCGACGAAGAAAGCTGGGACGAAGACGACGAAGAGTCCGAGGAAGCAGACGAAGACGGAGACTTCTAAGTGGAACTGCGGGAGAAAATCCTTGAGCGAATCAAGGTGACTTCCTCTGGGTGTTGGGAGTGGCAGGGCGCTACGAACAATAAAGGGTACGGGCAGGTGTGGTGCAGCAATACCGGAAAGGTTGTCTACTGTCATCGCGTAATGTCTAATGc                                                                                                                                                                                             <  1:569535/283‑1 (MQ=255)`  
                                                                                                                   `ggCGGTGAAGACCATTGGGATGACGAAGTTGAAGAGAACGGCTATGTTGCCTCTGGTTCAGCCAAAGAGAGCAAAACACGCGACGAAGAAAGCGGGGACGAAGACGACGAAGAGTCCGAGGAAGCAGACGAAGACGGAGACTTCTAAGTGGAACTGCGGGAGAAAATCCTTGAGCGAATCAAGGTGACTTCCTCTGGGTGTTGGGAGTGGCAGGGCGCTACGAACAATAAAGGGTACGGGCAGGTGTGGTGCAGCAATAACGGAAAGGTTGTCTACTGTCATCGCGTAATGTCTAATGCTc                                                                                                                                                                                           <  1:181171/301‑1 (MQ=255)`  
                                                                                                                         `gAAGACGATTGGGCTGACGAAGTTGAAGAGAACGGCTATGTTGCCTCTGGTTCTGCCAAAGCGAGCAAACCACGCGACGAAGAAAGCTGGGACGAAGACGACGAAGAGTCCGAGGAAGCAGACGAAGACGGAGACTTCTAAGTGGAACTGCGGGAGAAAATCCTTGAGCGAATCAAGGTGACTTCCTCTGGGTGTTGGGAGTGGCAGGGCGCTACGAACAATAAAGGGTACGGGCAGGTGTGGTGCAGCAATACCGGAAAGGTTGTCTACTGTCATCGCGTAATGTCTAATGCTCCGAAAg                                                                                                                                                                                     <  1:588745/301‑1 (MQ=255)`  
                                                                                                                          `aggaagATTGGGCTGACGAAGTTGAAGAGATCGGCTATGTTGCCTGTGGTTCTGCCAAAGCGAGCAAACCACGAGAAGAAAAAAGCTGGGACGAAGACGGCGAAGTGTCCGATGAAGCAGACGAAGGCGGAGACTTCTAAGTGGAACTGCGGGAGAAAATCCTTGAGCGAATCAAGGTGACTTCCTCTGGGTGTGGGGAGTGGCAGTGCGCTACGAACAATAAAGTGTACGGGCAGGTGTGGTGCAGCATTACCGGAAAGGTTGTCTACTGTCATCGCGTAATGGCTAATGCTCCAAAAgg                                                                                                                                                                                    <  1:411510/296‑1 (MQ=255)`  
                                                                                                                          `aaGACGATTGGGCTGACGAAGTTGAAGATAACGGCTATGTTGCCTCTGGTTCTGCCAAAGCGAGCAAACCACGCGACGAAGAAAGCTGGGACGAAGACGACGAAGAGTCCGAGGAAGCAGACGAAGACGGAGACTTCTAAGTGGAACTGCGGGAGAAAATCCTTGAGCGAATCAAGGTGACTTCCTCTGGGTGTTGGGAGTGGCAGGGCGCTACGAACAATAAAGGGTACGGGCAGGTGTGGTGCAGCAATACCGGAAAGGTTGTCTACTGTCATCGCGTAATGTCTAATGCTCCGAAAgg                                                                                                                                                                                    <  1:155214/301‑1 (MQ=255)`  
                                                                                                                                `gacaggCTGACGAAGTTGAAGAGAACGGCTATGTTGCCTCTGGTTCTGCCAAAGCGAGCAAACCACGCGACGAAGAAAGCTGGGACGAAGACGACGAAGAGTCCGAGGAAGCAGACGAAGACGGAGACTTCTAAGTGGAACTGCGGGAGAAAATCCTTGAGCGAATCAAGGTGACTTCCTCTGGGTGTTGGGAGTGGCAGGGCGCTACGAACAATAAAGGGTACGGGCAGGTGTGGTGCAGCAATACCGGAAAGGTTGTCTACTGTCATCGCGTAATGTCTAATGCTCCGAAAGGTTCTAc                                                                                                                                                                              <  1:478625/297‑1 (MQ=255)`  
                                                                                                                                    `ggCTGACGCAGTTGAAGAGAACGGCTATGTTGCCTCTGGTTCTGCCAAAGCGAGCAAACCACGCGACGAAGAAAGCTGGGACGAAGACGACGAAGAGACCGAGGACGCAGACGAAGACGGAGACTTCTAAGTGGAACTGCGGGAGAAAATCCTTGAGCGAATCAAGGTGACTTCCTCTGGGTGTTGGGAGTGGCAGGGCGCTACGAACAATAAAGGGTACGGGCAGGTGTGGTGCAGCAATACCGGAAAGGTTGTCTACTGTCATCGCGTAATGTCTAATGCTCCGAAAGGTTCTACCgcg                                                                                                                                                                          >  1:428782/1‑299 (MQ=255)`  
                                                                                                                                    `ggCTGACGAAGTTGAAGAGAACGGCTATGTTGCCTCTGGTTCTGCCAAAGCGAGCAAACCACGCGACGAAGAGAGCTGGGACGAAGACGACGAAGAGTCCGAGGAAGCAGACGAAGACGGAGACTTCTAAGTGGAACTGCGGGAGAAAATCCTTGAGCGAATCAAGGTGACTTCCTCTGGGTGTTGGGAGTGGCAGGGCGCTACGAACAATAAAGGGTACGGGCAGGTGTGGTGCAGCAATACCGGAAAGGTTGTCTACTGTCATCGCGTAATGTCTAATGCTCCGAAAGGTTCTACCGTc                                                                                                                                                                          >  1:339546/1‑301 (MQ=255)`  
                                                                                                                                        `gACGAAGTTGAAGAGAACGGCTATGTTGCCTCTGGTTCTGCCAAAGCGAGCAAACCACGCGACGAAGAAAGCTGGGACGAAGACGACGAAGAGTCCGAGGAAGCAGACGAAGACGGAGACTTCTAAGTGGAACTGCGGGAGAAAATCCTTGAGCGAATCAAGGTGACTTCCTCTGGGTGTTGGGAGTGGCAGGGAGCTACGAACAATAAAGGGTACGGGCAGGTGTGGTGCAGCAATACCGGAAAGGTTGTCTACTGTCATCGCGTAATGTCTAATGCTCCGAAAGGTTCTACCGTCCTGc                                                                                                                                                                      >  1:37548/1‑301 (MQ=255)`  
                                                                                                                                            `aaGTTGAAGAGAACGGCTATGTTGCCTCTGGTTCTGCCAAAGCGAGCAAACCACGCGACGAAGAAAGCTGGGACGAAGACGACGAAGAGTCCGAGGAAGCAGACGAAGACGGAGACTTCTAAGTGGAACTGCGGGAGAAAATCCTTGAGCGAATCAAGGTGACTTCCTCTGGGTGTTGGGAGTGGCAGGGCGCTACGAACAATAAAGGGTACGGGCAGGTGTGGTGCAGCAATACCGGAAAGGTTGTCTACTGTCATCGCGTAATGTCTAATGCTcctgtctcttatacacatctccgagc                                                                                                                                                                  >  1:260929/1‑277 (MQ=255)`  
                                                                                                                                            `aaGTTGAAGAGAACGGCTATGTTGCCTCTGGTTCTGCCAAAGCGAGCAAACCACGCGACGAAGAAAGCTGGGACGAAGACGACGAAGAGTCCGAGGAAGCAGACGAAGACGGAGACTTCTAAGTGGAACTGCGGGAGAAAATCCTTGAGCGAATCAAGGTGACTTCCTCTGGGTGTTGGGAGTGGCAGGGCGCTACGAACAATAAAGGGTACGGGCAGGGGTGGTGCAGCAATACCGGAAAGGTTGTATACTGTCATCGCGTAATGTCGAATGCTCCGAAAGGTTCTAcctgtctcttata                                                                                                                                                                  >  1:362857/1‑290 (MQ=255)`  
                                                                                                                                              `gTTGAAGAGAACGGCTATGTTGCCTCTGGTTCTGCCAAAGCGAGCAAACCACGCGACGAAGAAAGCTGGGACGAAGACGACGAAGAGTCCGAGGAAGCAGACGAAGACGGAGACTTCTAAGTGGAACTGCGGGAGAAAATCCTTGAGCGAATCAAGGTGACTTCCTCAGGGTGTTGGGAGTGGCAGGGCGCTACGAACAATAAAGGGTACGAGCAGGTGTGGTGCAGCAATACCGGAAAGGTTGTCTACTGTCATCGCGTAATGTCTAATGCTCCGAAAGGTTCTACCGTCCTGCACTCCt                                                                                                                                                                >  1:21876/1‑301 (MQ=255)`  
                                                                                                                                                  `aaGAGAACGGCTATGTTGCCTCTGGTTCTGCCAAAGCGAGCAAACCACGCGACGAAGAAAGCTGGGACGAAGACGACGAAGAGTCCGAGGAAGCAGACGAAGACGGAGACTTCTAAGTGGAGCTGCGGGAGAAAATCCTTGAGCGAATCAAGGTGACTTCCTCTGGGTGTTGGGAGTGGCAGGGCGCTACGAACAATAAAGGGTACGGGCAGGTGTGGTGCAGCAATACCGGAAAGGTTGTCTACTGTCATCGCGTAATGTCTAATGCTCCGAAAGGTTCTACCGTCCTGCACTCCTGTGa                                                                                                                                                            <  1:520530/301‑1 (MQ=255)`  
                                                                                                                                                    `gagaACGGCTATGTTGCCTCTGGTTCTGCCAAAGCGAGCAAACCACGCGACGAGGAAAGCTGGGACGAAGACGACGAAGAGTCCGAGGAAGCAGACGAAGACGGAGACTTCTAAGTGGAACTGCGGGAGAAAATCCTTGAGCGAATCAAGGTGACTTCCTCTGGGTGTTGGGAGTGGCAGGGCGCTACGAACAATAAAGGGTACGGGCAGGTGTGGTGCAGCAATACCGGAAAGGTTGTCTACTGTCATCGCGTAATGTCTAATGCTCCGAAAGGTTCTACCGTCCTGCACTCCTGTGATa                                                                                                                                                          >  1:32755/1‑301 (MQ=255)`  
                                                                                                                                                    `gagaACGGCTATGTTGCCTCTGGTTCTGCCAAAGCGAGCAAACCACGCGACGAAGAAAGCTGGGACGAAGACGACGAAGAGTCCGAGGAAGCAGACGAAGACGGAGACTTCTAAGTGGAACTGCGGGAGAAAATCCTTGAGCGAATCAAGGTGACTTCCTCTGGGTGTTGGGAGTGGCAGGGCGCTACGAACAATAAAGGGTACGGGCAGGTGTGGTGCAGCAATACCGGAAAGGTTGTCTACTGTCATCGCGTAATGTCTAATGCTCCGAAAGGTTCTACCGTCCTGCACTCCtgctgtc                                                                                                                                                          >  1:418407/1‑296 (MQ=255)`  
                                                                                                                                                       `acaggCTATGTTGCCTCTGGTTCTGCAAAAGCTAGCAAACCACGCGACGAAGAAAGCTGGGACGAAGACGACGAAGAGTCCGAGGAAGCAGACGAAGACGGAGACTTCTAAGTGGAACTGCGGGAGAAAATCCTTGAGGGAATCAAGGTGACTTCCTCTGGGTGTTGGGAGTGGCAGGGCGCTACGAACAATAAAGGGTACGGGCAGGTGTGGTGCAGCAATACCGGAAAGGTTGTCTACTGTCATCGCGTAATGTCTAATGCTCCGAAAGGTTCTACCGTCCTGCACTCCTGTGATAATc                                                                                                                                                       <  1:439131/298‑1 (MQ=255)`  
                                                                                                                                                       `aaCGGCTATGTTGCCTCTGGTTCTGCCAAAGCGAGCAAACCACGCGACGAAGAAAGCTGGGACGAAGACGACGAAGAGTCCGAGGAAGCAGACGAAGACGGAGACTTCTAAGTGGAACTGCGGGAGAAAATCCTTGAGCGAATCAAGGTGACTTCCTCTGTGTGTTGGGAGTGGCAGGGCGCTACGAACAATAAAGGGTACGGGCAGGTGTGGTGCAGCAATACCGGAAAGGTTGTCTACTGTCATCGCGTAATGTCTAATGCTCCGAAAGGTTCTACCGTCCTGCACTCCTGTGATAATc                                                                                                                                                       <  1:575505/301‑1 (MQ=255)`  
                                                                                                                                                         `cGGCTATGTTGCCTCTGGTTCTGCCAAAGCGAGCAAACCACGCGACGAAGAAAGCTGGGACGAAGACGACGAAGAGTCCGAGGAAGCAGACGAAGACGGAGACTTCTAAGTGGAACTTCGGGAGTAAATCCTTGAGCGAATCAAGGTGACTTCCTCTGGGTGTTGGGAGTGGCAGGGCGCTACGAACAATAAAGGGTACGGGCAGGTGTGGTGCAGCAATACCGGAAAGGTTGTCTACTGTCATCGCGTAATGTCTAATGCTCCGAAAGGTTCTACCGTCCTGCACTCCTGTGATAATCCa                                                                                                                                                     >  1:533914/1‑301 (MQ=255)`  
                                                                                                                                                           `gCTATGTTGCCTCTGGTTCTGCCAAAGCGAGCAAACCACGCGACGAAGAAAGCTGGGACGAAGACGACGAAGAGTCCGAGGAAGCAGACGAAGACGGAGACTTCTAAGTGGAACTGCGGGAGAAAATCCTTGAGCGAATCAAGGTGACTTCCTCTGGGTGTTGGGAGTGGCAGGGCGCTACGAACAATAAAGGGTACGGGCAGGTGTGGTGCAGCAATACCGGAAAGGTTGTCTACTGTCATCGCGTAATGTCTAATGCTCCGAAAGGTTCTACCGTCCTGCACTCCTGCGATAATCCAtt                                                                                                                                                   >  1:200249/1‑301 (MQ=255)`  
                                                                                                                                                              `aTGTTGCCTCTGGTTCTGCCAAAGCGAGCAAACCACGCGACGAAGAAAGCTGGGACGAAGACGACGAAGAGTCCGAGGAAGCAGACGAAGACGGAGACTTCTAAGTGGAACTGCGGGAGAAAATCCTTTAGCGAATCAAGGTGACTTCCTCTGGGTGTTGGGAGTGGCAGGGCGCTACGAACAATAAAGGGTACGGGCAGGTGTGGTGCAGCAATACCGGAAAGGTTGTCTACTGTCATCGCGTAATGTCTAATGCTCCGAAAGGTTCTACCGTCCTGCACTccctgtctcttctacacat                                                                                                                                                >  1:317117/1‑284 (MQ=255)`  
                                                                                                                                                              `aTGTTGCCTCTGGTTCTGCCAAAGCGAGCAAACCACGCGACGAAGAAAGCTGGGACGAAGACGACGAAGAGTCCGAGGAAGCAGACGAAGACGGAGACTTCTAAGTGGAACTGCGGGAGAAAATCCTTGAGCGAATCAAGGTGACTTCCTCTGGGTGTTGGGAGTGGCAGGTCGCTACGAACAATAAAGGGTACGGGCAGGTGTGGTGCAGCAATACCGGAAAGGTTGTCTACTGTCATCGCGTAATGTCTAATGCTCCGAAAGGTTCTACCGTCCTGCACTCCTGTGATAATCCATTAtg                                                                                                                                                <  1:292352/301‑1 (MQ=255)`  
                                                                                                                                                              `aTGTTGCCTCTGGTTCTGCCAAAGCGAGCAAACCACGCGACGAAGAAAGCTGGGACGAAGACGACGAAGAGTCCGAGGAAGCAGACGAAGACGGAGACTTCTAAGTGGAACTGCGGGAGAAAATCCTTGAGCGAATCAAGGTGACTTCCTCTGGGTGTTGGGAGTGGCAGGGCGCTACGAACAATAAAGGGTACGGGCAGGTGTGGTGCAGCAATATCGGAAAGGTTGTCTACTGTCATCGCGTAATGTCTAATGCTCCGAAAGGTTCTACCGTCCTGCACTccctgtctcttatacacat                                                                                                                                                >  1:595189/1‑284 (MQ=255)`  
                                                                                                                                                              `aTGTTGCCTCTGGTTCTGCCAAAGCGAGCAAACCACGCGACGAAGAAAGCTGGGACGAAGACGACGAAGAGTCCGAGGAAGCAGACGAAGACGGAGACTTCTAAGTGGAACTGCGGGAGAAAATCCTTGAGCGAATCAAGGTGACTTCCTCTGGGTGTTGGGAGTGGCAGGGCGCTACGAACAATAAAGGGTACGGGCAGGTGTGGTGCAGCAATACCGGAAAGGTTGTCTACTGTCATCGCGTAATGTCTAATGCTCCGAAAGGTTCTACCGTCCTGCACTCCTGTGATAATCCATTAtg                                                                                                                                                >  1:79299/1‑301 (MQ=255)`  
                                                                                                                                                               `taagagacagggTTCTGCCAAAGCGAGAAAACCACGCGACGAAGAAAGCTGGGACGAAGACGACGAAGAGGCCGAGGAAGCAGAAGAAGACGGAGACTTATAAGTGGAACTGCGGGAGAAAATCCTTGAGCGAATCAAGGTGACTTCCTCTGGGTGTGGGGAGTGGCAGGGCGCTACGAACAATAAAGGGTACGGGCAGGTGTGGTGCAGCAATACCGGAAAGGTTGTCTACTGTCATCGCGTAATGTCTAATGCTCCGAAAGGTTCTACCGTCCTGCACTCCTGTGATAATCCATTAtgt                                                                                                                                               <  1:395551/291‑1 (MQ=255)`  
                                                                                                                                                               `tGTTGACTCTGGTTCTGCCATAGCGAGCAAACCAGTCGACGAAGAAAGCTGGGACGAATACGACGAAGAGGCCTATGAAGCATACGAAGACGGAGACTTCTAAGTGGAACTGCGGTAGAAAATCCTTGAGCGAATCAAGGTGACTGCCTCTGGGTGTTGGGATTGGCAGGGCGCTACGAACAATAAAGGGTACTGGCATGTGTGGTGCATCAATACCGGAAAGGTTGTCTACTGTCATCGCGTAATGTCTAATGCTCCGAAAGGTTCTACCGTCCTGCACTCCTGTGATAATCCATTAtgt                                                                                                                                               <  1:142917/301‑1 (MQ=255)`  
                                                                                                                                                               `acaggCCTCTGGTTCTGCCAAAGCGAGCAAACCACGCGACGAAGAAAGCTGGGACGAAGACGACGAAGAGTCCGAGGAAGCAGACGAAGACGGAGACTTCTAAGTGGAACTGCGGGAGAAAATCCTTGAGCGAATCAAGGTGACTTCCTCTGGGTGTTGGGAGTGGCAGGGCGCTACGAACAATAAAGGGTACGGGCAGGTGTGGTGCAGCAATACCGGAAAGGTTGTCTACTGTCATCGCATAATGTCTAATGCTCCGAAAGGTTCAACCGTCCTGCACTCCTGTGATAATCCATTAtgt                                                                                                                                               <  1:45138/297‑1 (MQ=255)`  
                                                                                                                                                                 `ttGCCTCTGGTTCCGCCAAAGCGAGCAAACCACGCGATGAAGAAAGCTGGGACGAAGACGACGAAGAGTCCGAGGAAGCAGACGAAGACGGAGACTTCTAAGTGGAACTGCGGGAGAAAATCCTTGAGCGAATCAAGGTGACTTCCTCTGGGTGTTGGGAGTGGCAGGTCGCTACGAACAATAAAGGGTACGGGCAGGTGTGGTGCAGCAATACCGGAAAGGTTGTCTACTGTCATCGCGTAATGTCTAATGCTCCGAAAGGTTCTACCGTCCTGCACTCCTGTGATAATCCATTAtgttg                                                                                                                                             <  1:206082/301‑1 (MQ=255)`  
                                                                                                                                                                       `ctGGTTCTGCCAAAGCGAGCAAACCACGCGACGAAGAAAGCTGGGACGAAGACGACGAAGAGTCCGAGGAAGCAGACGAAGACGGAGACTTTTAAGTGGAACTGCGGGAGAAAATCCTTGAGCGAATCAAGGTGACTTCCTCTGGGTGTTGGGAGTGGCAGGGCGCTACGAACAATAAAGGGTACGGGCAGGTGTGGTGCAGCAATACCGGAAAGGTTGTCTACTGTCATCGCGTAATGTCTAATGCTCCGAAAGGTTCTACCGTCCTGCACTCCTGTGATAATCCATTATGTTGTAAccc                                                                                                                                       <  1:249047/301‑1 (MQ=255)`  
                                                                                                                                                                       `ctGGTTCTGCCAAAGCGAGCAAACCACGCGACGAAGAAAGCTGGGACGAAGACGACGAAGAGTCCGAGGAAGCAGACGAAGACGGAGACTTCTAAGTGGAACTGCGGGAGAAAATCCTTGAGCGAATCAAGGTGACTTCCTCTGGGTGTTGGGAGTGGCAGGGCGCTACGAACAATAAAGGGTACGGGCAGGTGTGGTGCAGCAATACCGGAAAGGTTGTCTACTGTCATCGCGTAATGTCTAATGCTCCGAAAGGTTCTACCGTCCTGCACTCCTGTGATAATCCATTATGTTGTAAccc                                                                                                                                       <  1:601117/301‑1 (MQ=255)`  
                                                                                                                                                                       `ctGGTTCTGCCAAAGCGAGCAAACCACGCGACGAAGAAAGCTGGGACGAAGAAGACGAAGAGTCCGAGGAAGCAGACGAAGACGGAGACTTCTAAGTGGAACTGCGGGAGAAAATCCTTGAGCGAATCAAGGTGACTTCCTCTGGGTGTTGGGAGTGGCAGGGCGCTACGAACAATAAAGGGTACGGGCAGGTGTGGTGCAGCAATACCGGAAAGGTTGTCTACTGTCATCGCGTAATGTCTAATGCTCCGAAAGGTTCTACCGCCCTGCACTCCTGTGATAATCCATTATGTTGTAAccc                                                                                                                                       <  1:272048/301‑1 (MQ=255)`  
                                                                                                                                                                             `cTGCCAAAGCGAGCAAACCACGCGACGAAGAAAGCTGGGACGAAGACGACGAAGAGTCCGAGGAAGCAGACGAAGACGGAGACTTCTAAGTGGAACTGCGGGAGAAAATCCTTGAGCGAATCAAGGTGACTTCCTCTGGGTGTTGGGAGTGGCAGGGCGCTACGAACAATAAAGGGTACGGGCAGGTGTGGTGCAGCAATACCGGAAAGGTTGTCTACTGTCATCGCGTAATGTCTAATGCTCCGAAAGGTTCTACCGTCCTGCACTCCTGTGATAATCCATTATGTTGTAACCCTGAaca                                                                                                                                 <  1:255169/301‑1 (MQ=255)`  
                                                                                                                                                                                    `aGCGAGCAAACCACGCGACGTAGAAAGCTGGGACGAAGACGACGAAGAGTCCGAGGAAGCAGACGAAGACGGAGACTTCTAAGTGGAACTGCGGGAGAAAATCCTTGAGCGAATCAAGGTGACTTCCTCTGGGTGTTGGGAGTGGCAGGGCGCTACGAACAATAAAGGGTACGGGCAGGTGTGGTGCAGCAATACCGGAAAGGTTGTCTACTGTCATCGCGTAATGTCTAATGCTCCGAAAGGTTCTACCGTCCTGCACTCCTGTGATAATACATTATGTTGTAACCCTGAACACCTATcc                                                                                                                          <  1:67174/301‑1 (MQ=255)`  
                                                                                                                                                                                          `cAAACCACGCGACGAAGAAAGCTGGGACGAAGACGACGAAGAGTCCGAGGAAGCAGACGAAGACGGAGACTTCTAAGTGGAACTGCGGGAGAAAATCCTTGAGCGAATCAAGGTGACTTCCTCTGGGTGTTGGGAGTGGCAGGGCGCTACGAACAATAAAGGGTACGGGCAGGTGTGGTGCAGCAATACCGGAAAGGTTGTCTACTGTCATCGCGTAATGTCTAATGCTCCGAAAGGTTCTACCGTCCTGCACTCCTGTGATAATCCATTATGTTGTAACCCTGAACACCTATCCATAGGa                                                                                                                    <  1:267421/301‑1 (MQ=255)`  
                                                                                                                                                                                                       `gaagaaAGCTGGGACGAAGACGACGAAGAGTCCGAGGAAGCAGACGAAGACGGAGACTTCTAAGTGGAACTGCGGGAGAAAATCCTTGAGCGAATCAAGGTGACTTCCTCTGGGTGTTGGGAGTGGCAGGGCGCTACGAACAATAAAGGGTACGGGCAGGTGTGGTGCAGCAATACCGGAAAGGTTGTCTACTGTCATCGCGTAATGTCTAATGCTCCGAAAGGTTCTACCGTCCTGCACTCCTGTGATAATCCATTATGTTGTAACCCTGAACACCTATCCATAGGAACTCCAAAAgaga                                                                                                       >  1:633941/1‑301 (MQ=255)`  
                                                                                                                                                                                                       `gaagaaAGCTGGGACGAAGACGACGAAGAGTCCGAGGAAGCAGACGAAGACGGAGACTTCTAAGTGGAACTGCGGGAGAAAATCCTTGAGCGAATCAAGGTGACTTCCTCTGGGTGTTGGGAGTGGCAGGGCGCTACGAACAATAAAGGGTACGGGCAGGTGTGGTGCAGCAATACCGGAAAGGTTGTCTACTGTCATCGCGTAATGTCTAATGCTCCGAAAGGTTCTACCGTCCTGCACTCCTGTGATAATCCATTATGTTGTAACCCTGAACACCTATCCATAGGAACTCCAAAAgaga                                                                                                       >  1:100175/1‑301 (MQ=255)`  
                                                                                                                                                                                                          `gaaAGCTGGGACGAAGACGACGAAGAGTCCGAGGAAGCAGACGAAGACGGAGACTTCTAAGTGGAACTGCGGGAGAAAATCCTTGAGCGAATCAAGGTGACTTCCTCTGGGTGTTGGGAGTGGCAGGGCGCTACGAACAATAAAGGGTACGGGCAGGTGTGGTGCAGCAATACCGGAAAGGTTGTCTACTGTCATCGCGTAATGTCTAATGCTCCGAAAGGTTCTACCGTCCTGCACTCCTGTGATAATCCATTATGTTGTAACCCTGAACACCTATCCATAGGAACTCCAAAAGAGAACt                                                                                                    >  1:563640/1‑301 (MQ=255)`  
                                                                                                                                                                                                                 `gggACGAAGACGACGAAGAGTCCGATGAAGCAGACGAAGACGGAGACTTCTAAGTGGAACTGCGGGAGAAAATCCTTGAGCGAATCAAGGTGACTTCCTCTGGGTGTTGGGAGTGGCAGGGCGCTACGAACAATAAAGGGTACGGGCAGGTGTGGTGCAGCAATACCGGAAAGGTTGTCTACTGTCATCGCGTAATGTCTAATGCTCCGAAAGGTTCTACCGTCCTGCACTCCTGTGATAATCCATTATGTTGTAACACTGAACACCTATCCATAGGAACTCCAAAAGAGAACTCCACTGa                                                                                             <  1:23178/301‑1 (MQ=255)`  
                                                                                                                                                                                                                  `ggACGAAGACGACGAAGAGTCCGAGGAAGCAGACGAAGACGGAGACTTCTAAGTGGAACTGCGGGAGAAAATCCTTGAGCGAATCAAGGTGACTTCCTCTGGGTGTTGGGAGTGGCAGGGCGCTACGAACAATAAAGGGTACGGGCAGGTGTGGTGCAGCAATACCGGAAAGGTTGTCTACTGTCATCGCGTAATGTCTAATGCTCCGAAAGGTTCTACCGTCCTGCACTCCTGTGATAATCCATTATGTTGTAACCCTGAACACCTATCCATAGGAACTCCAAAAGAGAACTCCACTGAc                                                                                            >  1:508889/1‑301 (MQ=255)`  
                                                                                                                                                                                                                        `accagacgaAGAGTCCGAGGAAGCAGACGAAGACGGAGACTTCTAAGTGGAACTGCGGGAGAAAATCCTTTAGCGAATCAAGGTGACTTCCTCTGGGTGTTGGGAGTGGCAGGGCGCTACGAACAATAAAGGGTACGGGCAGGTGTGGTGCAGCAATACCGGAAAGGTTGTCTACTGTCATCGCGTAATGTCTAATGCTCCGAAAGGTTCTACCGTCCTGCACTCCTGTGATAATCCATTATGTTGTAACCCTGAACACCTATCCATAGGAACTCCAAAAGAGAACTCCACTGACATGGTa                                                                                      <  1:665319/297‑1 (MQ=255)`  
                                                                                                                                                                                                                         `gacgacGAAGAGTCCGAGGAAGCATACGAAGACGGAGACTTCTAAGTGGAACTGCGGGAGAAAATCCTTGAGCGAATCAAGGTGACTTCCTCTGGGTGTTGGGAGTGGCAGGGCGCTACGAACAATAAAGGGTAC‑GGCAGGTGTGGTGCAGCAATACCGGAAAGGTTGTCTACTGTCATCGCGTAATGTCTAATGCTCCGAAAGGTTCTACCGTCCTGCACTCCTGTGATAATCCATTATGTTGTAACCCTGAACACCTATCCATAGGAACTCCAAAAGAGAACCCCACTGACATGGTaaa                                                                                    >  1:162240/1‑301 (MQ=255)`  
                                                                                                                                                                                                                             `gagacaggTCCGAGGAAGCAGATGAAGACGGAGACTTCTAAGTGGAACTGCGGGAGAAAATCCTTGAGCGAATCAAGGTGACTTCCTCTGGGTGTTGGGAGTGGCAGGGCGCTACGAACAATAAAGGGTACGGGCAGGTGTGGCGCAGCAATACCGGAAAGGTTGTCTACTGTCATCGCGTAATGTCTAATGCTCCGAAAGGTTCTACCGTCCTGCACTCCTGTGATAATCCATTATGTTGTAACCCTGAACACCTATCCATAGGAACTCCAAAAGAGAACTCCACTGACATGGTAAATaa                                                                                 <  1:629395/294‑1 (MQ=255)`  
                                                                                                                                                                                                                             `gacaggagTCCGAGGAAGCAGACGAAGACGGAGACTTCTAAGTGGAACTGCGGGAGAAAATCCTTGAGCGAATCAAGGTGACTTCCTCTGGGTGTTGGGAGTGGCAGGGCGCTACGAACAATAAAGGGTACGGGCAGGTGTGGTGCAGCAATACCGGAAAGGTTGTCTACTGTCATCGCGTAATGTCTAATGCTCCGAAAGGTTCTACCGTCCTGCACTCCTGTGATAATCCATTATGTTGTAACCCTGAACACCTATCCATAGGAACTCCAAAAGAGAACTCCACTGACATGGTAAATaa                                                                                 <  1:489630/296‑1 (MQ=255)`  
                                                                                                                                                                                                                             `acgaAGAGTCCGAGGAAGCAGACGAAGACGGAGACTTCTAAGTGGAACTGCGGGAGAAAATCCTTGAGCGAATCAAGGTGACTTCCTCTGGGTGTTGGGAGTGGCAGGGCGCTACGAACAATAAAGGGTACGGGCAGGTGTGGTGCAGCAATACCGGAAAGGTTGTCTACTGTCATCGCGTAATGTCTAATGCTCCGAAAGGTTCTACCGTCCTGCACTCCTGTGATAATCCATTATGTTGTAACCCTGAACACCTATCCATATGAACTCCAAAAGAGAACTCCACTGACATGGTAAATaa                                                                                 <  1:495431/301‑1 (MQ=255)`  
                                                                                                                                                                                                                             `acgaAGAGTCCGAGGAAGCAGACGAAGACGGAGACTTCTAAGTGGAACTGCGGGAGAAAATCCTTGAGCGAATCAAGGTGACTTCCTCTGGGTGTTGGGAGTGGCAGGGCGCTACGAACAATAAAGGGTACGGGCAGGTGTGGTGCAGCAATACCGGAAAGGTTGTCTACTGTCATCGCGTAATGTCTAATGCTCCGAAAGGTTCTACCGTCCTGCACTCCTGTGATAATCCATTATGTTGTAACCCTGAACACCTATCCATAGGAACTCCAAAAGAGAACTCCACTGACATGGTAAATaa                                                                                 <  1:514828/301‑1 (MQ=255)`  
                                                                                                                                                                                                                             `acgaAGAGTCCGAGGAAGCAGACGAAGACGGAGACTTCTAAGTGGAACTGCGGGAGAAAATCCTTGAGCGAATCAAGGTGACTTCCTCTGGGTGTTGGGAGTGGCAGGGCGCTACGAACAATAAAGGGTACGGGCAGGTGTGGTGCAGCAATACCGGAAAGGTTGTCTACTGTCATCGCGTAATGTCTAATGCTCCGAAAGGTTCTACCGTCCTGCACTCCTGTGATAATCCATTATGTTGTAACCCTGAACACCTATCCATAGGAACTCCAAAAGAGAACTCCACTGACATGGTAAATaa                                                                                 <  1:607767/301‑1 (MQ=255)`  
                                                                                                                                                                                                                              `cgaAGAGTCCGAGGAAGCAGACGAAGACGGAGACGTCTAAGTGGAACTGCGGGAGAAAATCCTTGAGCGAATCAAGGTGACTTCCTCTGGGTGTTGGGAGTGGCAGGGCGCTACGAACAATAAAGGGTACGGGCAGGTGTGGTGCAGCAATACCGGAAAGGTTGTCTACTGTCATCGCGTAATGTCTAATGCTCCGAAAGGTTCTACCGTCCTGCACTCCTGTGATAATCCATTATGTTGTAACCCTGAACACCTATCCATAGGAACTCCAAAAGAGAACTCCACTGACATGGTAAATAAg                                                                                <  1:299509/301‑1 (MQ=255)`  
                                                                                                                                                                                                                                            `gaGCAGACGAAGACAGAGAATTCTAAATGGAAATGCGGAGGAAAATCCAAGAGCGAATCAAGGAGACTTCCTCTGGGTGGTGGGGGTGGCAGGGAGCTACGAACAATAAAGGGTACGGGCAAGTGTGGGGCAGCAATACCGGAAAGGTTGTCTACTGTCATCGCGTAATGTCTAATGCTCCGAAAGGTTCTACCGTCCTGCACGCCTGTGATAATCCATTATGTTGTAACCCTGAACACCTATCCATAGGAAAGCCAAAAGAGAACGCCACTGACATGGTAAATAAGGGTCGCTCACACaa                                                                  <  1:22542/300‑1 (MQ=255)`  
                                                                                                                                                                                                                                              `gCAGACGAAGACGGAGACTTCTAAGTGGAACTGCGGGAGAAAATCCTTGAGCGAATCAAGGTGACTTCCTCTGGGTGTTGGGAGTGGCAGGGCGCTACGAACAATAAAGGGTACGGGCAGGTGTGGTGCAGCAATACCGGAAAGTTTGTCTACTGTCATCGCGTAATGTCTAATGCTCCGAAAGGTTCTACCGTCCTGCACTCCTGTGCTAATCCATTATGTTGTAACCCTGAACACCTATCCATAGGAACTCCAAAAGAGAACTCCACTGACCTgtctcttataaccatctccgagcccc                                                                >  1:311619/1‑276 (MQ=255)`  
                                                                                                                                                                                                                                                `aacagAAGATGGAGACTTCTAAGTGGAACTGCGGGAGAAAATCCTTGAGCGAATCAAGGTGACTTCCTCTGGGTGTTGGGAGTGGCAGGGCGCTACGAACAATAAAGGGTACGGGCAGGTGTGGTGCAGCAAGACCGGAAAGGTTGTCTACTGTCATCGCGTAATGTCTAATGCTCCGAAAGGTTCTACCGTCCTGCACTCCTGTGATAATCCATTATGTTGTATCCCTGAACACCTATCCATAGGAACTCCAAAAGAGAACTCCACTGACATGGTAAATAAGGGTCGCTCACACAAgggg                                                              <  1:110847/297‑1 (MQ=255)`  
                                                                                                                                                                                                                                                 `gACGAAGACGGAGACTTCTAAGTGGAACTGCGGGAGAAAATCCTTGAGCGAATCAAGGTGACTTCCTCTGGGTGTTGGGAGTGGCAGGGCGCTACGAACAATAAAGGGTACGGGCAGGTGTGGTGCAGCAATACCGGAAAGGTTGTCTACTGTCATCGCGTATTGTCTAATGCTCCGAAAGGTTCTACCGTCCTGCACTCCTGTGATAATCCATTATGTTGTAACCCTGAACACCTATCCATAGGAACTCCAAAAGAGAACTCCACTGACATGGTAAATAAGGGTCGCTCACAAAAGGGGt                                                             >  1:457297/1‑301 (MQ=255)`  
                                                                                                                                                                                                                                                 `gACGAAGACGGAGACTTCTAAGTGGAACTGAGGGAGAAAATCCTTGAGCGAATCAAGGTGACTTCCTCTGGGTGTTGGGAGTGGCAGGGCGCTACGAACAATAAAGGGTACGGGCAGGTGTGGTGCAGCAATACCGGAAAGGTTGTCTACTGTCATCGCGTAATGTCTAATGCTCCGAAAGGGTCTACCGTCCTGCACTCCTGTGATAATCCATTATGTTGTAACCCTGAACACCTATCCATAGGAACTCCAAAAGAGAACTCAACTGACATGGAAAATAAgctgtcacttataaacatct                                                             >  1:350537/1‑282 (MQ=255)`  
                                                                                                                                                                                                                                                           `gagaCTTCTAAGTGGAACTGCGGGAGAAAATCCTTGAGCGAATCAAGGTGACTTCCTCTGGGTGTTGGGAGTGGCAGGGCGCTACGAACAATAAAGGGTACGGGCAGGTGTGGTGCAGCAATACCGGAAAGGTTGTCTACTGTCATCGCGTAATGTCTAATGCTCCGAAAGGTTCTACCGTCCTGCACTCCTGTGATAATCCATTATGTTGTAACCCTGAACACCTATCCATAGGAACTCCAAAAGAGAACTCCACTGACATGGTAAATAAGGGTCGCTCACACAAGGGGTATAAACTTTc                                                   >  1:11660/1‑301 (MQ=255)`  
                                                                                                                                                                                                                                                               `cTTCTAAGTGGAACTGCGGGAGAAAATCCTTGAGCGAATCAAGGTGACTTCCTCTGGGTGTTGGGAGTGGCAGGGCGCTACGAACAATAAAGGGTACGGGCAGGTGTGGTGCAGCAATACCGGAAAGGTTGTCTACTATCATCGCGTAATGTCTAATGCTCCGAAAGGTTCTACCGTCCTGCACTCCTGTGATAATCCATTATGTTGTAACCCTGAACACCTATCCATAGGAACTCCAAAAGAGAACTCCACTGACATGGTAAATAAGGGTCGCTCACACAAGGGGTATAAACTTTCAGAc                                               >  1:285768/1‑301 (MQ=255)`  
                                                                                                                                                                                                                                                                      `gTGGAACTGCGGGAGAAAATCCTTGAGCGAATCAAGGTGACTTCCTCTGGGTGTTGGGAGTGGCAGGGCGCTACGAACAATAAAGGGTACGGGCAGGTGTGGTGAAGCAATACCGGAAAGGTTGTCTACTGTCATCGAGTAATGTCTAATGCTCCGAAAGGTTCTACCGTCCTGCACTCCAGTGATAATCCATTATGTTGTAACCCTGAACACCTATCCATAGGAACTACAAAAGAGAACTCCTCTGACCTGGTAAATAAGGGTCGCTCACACAAGGGGTATAAACTTTCAGACGAAGACg                                        >  1:279351/1‑301 (MQ=255)`  
                                                                                                                                                                                                                                                                                 `ggAGAAAATCCTTGAGCGAATCAAGGTGACTTCCTCTGGGTGTTGGGAGTGGCAGGGCGCTACGAACAATAAAGGGTACGGGCAGGTGTGGTGCAGCAATACCGGAAAGGTTGTCTACTGTCATCGCGTAATGTCTAATGCTCCGAAAGGTTCTACCGTCCTGCACTCCTGTGATAATCCATTATGTTGTAACCCTGAACACCTATCCATAGGAACTCCAAAAGAGAACTCCACTGACATGGTAAATAAGGGTCGCTCACACAAGGGGTATAAACTTTCAGACGAAGACGTAATGGCAATc                             >  1:406271/1‑301 (MQ=255)`  
                                                                                                                                                                                                                                                                                          `ccTTGAGAGAATCAAGGTGACTTCCTCTGGGTGTTGGGAGTGGCAGGGCGCTACGAACAATAAAGGGTACGGGCAGGTGTGGTGCAGCAATACCGGAAAGGTTGTCTACTGTCATCGCGTAATGTCTAATGCTCCGAAAGGTTCTACCGTCCTGCACTCCTGTGATAATCCATTATGTTGTAACCCTGAACACCTATCCATAGGAACTCCAAAAGAGAACTCCACTGACATGGTAAATAAGGGTCGCTCACACAAGGGGTATAAACTTTCAGACGAAGACGTAATGGCAATCATGGAGTcc                    <  1:94998/301‑1 (MQ=255)`  
                                                                                                                                                                                                                                                                                                      `cAAGGTGACTTCCTCTGGGTGTTGGGAGTGGCAGGGCGCTACGAACAATAAAGGGTACGGGCAGGTGTGGTGCAGCAATACCGGAAAGGTTGTCTACTGTCATCGCGTAATGTCTAATGCTCCGAAAGGTTCTACCGTCCTGCACTCCTGTGATAATCCATTATGTTGTAACCCTGAACACCTATCCATAGGAACTCCAAAAGAGAACTCCACTGACATGGTAAATAAGGGTCGCTCACACAAGAGGTATAAACTTTCAGACGAAGACGTAATGGCAATCATGGAGTCCCGCGAGTCCact        >  1:188302/1‑299 (MQ=255)`  
                                                                                                                                                                                                                                                                                                          `gTGACTTCCTCTGGGTGTTGGGAGTGGCAGGGCGCTACGAACAATAAAGGGTACGGGCAGGTGTGGTGCAGCAATACCGGAAAGGTTGTCTACTGTCATCGCGTAATGTCTAATGCTCCGAAAGGTTCTACCGTCCTGCACTCCTGTGATAATCCATTATGTTGTAACCCTGAACACCTATCCATAGGAACTCCTAAAGAGAACTCCACTGACATGGTAAATAAGGGTCGCTCACACAAGGGGTATAAACTTTCAGACGAAGACGTAATGGCAATCATGGAGTCCAGCGAGTCCAATGTAt    >  1:423708/1‑301 (MQ=255)`  
                                                                                                                                                                                                                                                                                                            `gACTTCCTCTGGGTGTTGGGATGGGCAGGGCGCTACGAACAATAAAGGGTACGGGCAGGTGTGGTGCAGCAATACCGGAAAGGTTGTCTACTGTCATCGCGTAATGTCTAATGCTCCGAAAGGTTCTACCGTCCTGCACTCCTGTGATAATCCATTATGTTGTAACCCTGAACACCTATCCATAGGAACTCCAAAAGGGAACTCCACTGACATGGTAAATAAGGGTCGCTCACACAAGGGGTATAAACTTTCAGACGAAGACGTAATGGCAATCATGGAGTCCAGCGAGTCCAATGTATcc  <  1:382889/301‑1 (MQ=255)`  

                                                                                                                                                                                                                                                                                                            `|`                                                                                                                                                                                                                                                                                                              
`CTCTAAGCTGAAAGTTAAATATTCTCTGGTTCCATACAAGTGGAACACTGCTGTAGGTGCGAGCGTTAAGCTGCAACTGGAATCCGTGATGCTGGTCGAACTGGCTACCTTTGGTGGCGGTGAAGACGATTGGGCTGACGAAGTTGAAGAGAACGGCTATGTTGCCTCTGGTTCTGCCAAAGCGAGCAAACCACGCGACGAAGAAAGCTGGGACGAAGACGACGAAGAGTCCGAGGAAGCAGACGAAGACGGAGACTTCTAAGTGGAACTGCGGGAGAAAATCCTTGAGCGAATCAAGGTGACTTCCTCTGGGTGTTGGGAGTGGCAGGGCGCTACGAACAATAAAGGGTACGGGCAGGTGTGGTGCAGCAATACCGGAAAGGTTGTCTACTGTCATCGCGTAATGTCTAATGCTCCGAAAGGTTCTACCGTCCTGCACTCCTGTGATAATCCATTATGTTGTAACCCTGAACACCTATCCATAGGAACTCCAAAAGAGAACTCCACTGACATGGTAAATAAGGGTCGCTCACACAAGGGGTATAAACTTTCAGACGAAGACGTAATGGCAATCATGGAGTCCAGCGAGTCCAATGTATCC  >  NC_001604.1/9595‑10195`

  

Alignment Legend

Aligned base mismatch/match (shaded by quality score): `ATCG/ATCG` `< 3 ≤` `ATCG/ATCG` `< 10 ≤` `ATCG/ATCG` `< 23 ≤` `ATCG/ATCG` `< 34 ≤` `ATCG/ATCG` `< 38 ≤` `ATCG/ATCG`

Unaligned base: `atcg`    Masked matching base: `atcg`    Alignment gap: `‑`    Deleted base: `‑`

Reads not counted as support for junction

read\_name Not counted due to insufficient overlap past the breakpoint.

read\_name Not counted due to not crossing MOB target site duplication.
